# Increased CK5/CK8-Positive Intermediate Cells with Stromal Smooth Muscle Cell Atrophy in the Mice Lacking Prostate Epithelial Androgen Receptor

**DOI:** 10.1371/journal.pone.0020202

**Published:** 2011-07-06

**Authors:** Yuanjie Niu, Juan Wang, Zhiqun Shang, Shu-Pin Huang, Chih-Rong Shyr, Shuyuan Yeh, Chawnshang Chang

**Affiliations:** 1 Chawnshang Chang Sex Hormone Research Center, Tianjin Institute of Urology, Tianjin Medical University, Tianjin, China; 2 George Whipple Lab for Cancer Research, Departments of Pathology and Urology, University of Rochester Medical Center, Rochester, New York, United States of America; 3 Sex Hormone Research Center, China Medical University and Hospital, Taichung, Taiwan; Florida International University, United States of America

## Abstract

Results from tissue recombination experiments documented well that stromal androgen receptor (AR) plays essential roles in prostate development, but epithelial AR has little roles in prostate development. Using cell specific knockout AR strategy, we generated pes-ARKO mouse with knock out of AR only in the prostate epithelial cells and demonstrated that epithelial AR might also play important roles in the development of prostate gland. We found mice lacking the prostate epithelial AR have increased apoptosis in epithelial CK8-positive luminal cells and increased proliferation in epithelial CK5-positive basal cells. The consequences of these two contrasting results could then lead to the expansion of CK5/CK8-positive intermediate cells, accompanied by stromal atrophy and impaired ductal morphogenesis. Molecular mechanism dissection found AR target gene, TGF-β_1_, might play important roles in this epithelial AR-to-stromal morphogenesis modulation. Collectively, these results provided novel information relevant to epithelial AR functions in epithelial-stromal interactions during the development of normal prostate, and suggested AR could also function as suppressor in selective cells within prostate.

## Introduction

The prostate arises from the endodermal epithelium of the urogenital sinus (UGS), which is surrounded by an embryonic connective tissue called the urogenital sinus mesenchyme (UGM). In response to testosterone secreted from the fetal testis, epithelial buds emerge from the wall of the UGS and grow into the surrounding UGM, and undergo branching morphogenesis in the perinatal period. Beginning at the neonatal period, the epithelial cords undergo ductal canalization during which the epithelial cells differentiate into the luminal and basal cells, accompanied by differentiation of the mesenchyme into smooth muscle cells and fibroblasts [1]. Furthermore, a close reciprocal interaction between the epithelial and stromal tissue components may also play important roles for development of the prostate.

In the prostate, TGF-β signals may play important roles in the differentiation of prostate stroma by stimulation of the mature of smooth muscle cells. TGF-β and TGF-β receptors are expressed both in the epithelium and stroma of prostate [2]–[4]. It has become increasingly apparent that TGF-β intimately regulates the proliferation, growth arrest, and differentiation of human prostatic stromal cells, which is increased in benign prostatic hyperplasia (BPH) [5]–[7]. Previous studies also reported that TGF-β is an important regulator of stromal cell growth and promote the differentiation of prostatic stromal cells towards smooth muscle cell phenotype [8]–[10]. Early reports also speculated the existence of cross-talk mechanisms between androgen/AR and TGF-β signaling in benign and malignant prostate disease [11], [12]. But, how epithelial AR regulates the expression and secretion of epithelium TGF-β has not been reported.

Although both epithelial cells and stromal cells contain AR and the epithelial cells produce much larger amounts of dihydrotestosterone (DHT). Using tissue recombination strategy, Cunha *et al* demonstrated that stromal AR, but not epithelial AR, might play essential roles for the prostate development [13]–[16]. However, using cre-loxP strategy to knockout AR in epithelium, we found here that loss of epithelial AR could lead to the loss of the functional luminal cells, expanded progenital cell population, impaired ductal morphogenesis, impaired smooth muscle differentiation, and decreased epithelium-derived TGF-β_1_ expression. Together, these data suggest that epithelial AR may play important roles for the differentiation of prostate epithelium and the maturation of prostate stroma.

## Methods

### Cell culture

We maintained human prostate cancer cell lines in RPMI 1640 media with 10% fetal calf serum, 25 Units/ml penicillin, and 25 µg/ml streptomycin.

### Light Microscopy Procedures

Tissue samples were fixed in 5% neutral buffered formalin, embedded in paraffin, and cut into 5-µm thick slide sections. After H&E or immunostaining, we first identified the desired area by light microscopy using a low power dry objective lens. We then placed a small drop of oil on the coverslip for oil immersion lens high magnification and high resolution (×1,000) images of area. We counted the percentage of the positive cells and results were averaged from at least five different viewing areas.

### Generation of prostatic epithelium specific AR knockout (pes-ARKO) mouse

To generate pes-ARKO mice, we mated ARRPB2-Cre transgenic mice (C57BL/6) with mice containing the conditional AR allele (floxed AR, C57BL/6). Probasin Cre (Pb-Cre) (C57BL/6) mice were obtained from NCI. The genotype of ARKO mice was confirmed by PCR screening using mouse tail snip DNA. The deletion of AR exon2 was further confirmed by RT-PCR amplifying AR mRNA from mouse prostate using exon1 and exon3 primers.

### RNA Extraction, RT-PCR, and Real-Time RT-PCR

We harvested tissues or cultured cells in TRIzol (Invitrogen) and extracted total RNA following the manufacturer's instructions. We reverse transcribed (RT) 5 µg total RNA into 20 µl cDNA by the SuperScript III kit (Invitrogen) with oligo(dT) primer. The 20 µl cDNA was then diluted by water into 200 µl. Two µl reverse transcribed cDNA were used for PCR and real-time quantitative PCR the MyCycler thermal cycler (Bio-RAD) with by Taq polymerase and on the iCycler IQ multicolor real-time PCR detection system with 1/5 µl cDNA amplified by SYBR Green PCR Master Mix, respectively. We designed primers by Beacon Designer 2 software and used the β-actin expression level as control to calculate the relative gene expression among different samples. We calculated ¤ threshold (CT) values by subtracting the control CT value from the corresponding β-actin CT at each time point. We confirmed the absence of nonspecific amplification products by agarose-gel electrophoresis.

### Immunohistochemistry

We fixed samples in 5% neutral buffered formalin and embedded in paraffin. We used the primary antibodies of the rabbit anti-Ki67 (Abcam), the rabbit anti-Tag (Santa Cruz), the rabbit anti-AR (C19) (Santa Cruz Biotechnology), anti-CK5 (Covance), anti-CK8 (Abcam), anti-CD44 (Cell Signaling), anti-TGF-β1 (Santa Cruz), and anti-pSmad2/3 (Santa Cruz). The primary antibody was recognized by the biotinylated secondary antibody (Vector), and visualized by VECTASTAIN ABC peroxidase system (Vector) and peroxidase substrate DAB kit (Vector). The positive stainings were semi-quantitated by Image J software.

### Immunofluorescence Staining of CK5 and CK8 in Mouse Prostate Tumors

Tissue sections were incubated overnight at 4°C with primary antibodies, mouse anti-CK5 (Covance), and chicken anti-CK8 antibody (Abcam). After a 60-min rinse (3×20 min, PBS 1% Triton-X 100), we incubated slides with secondary antibodies (Alexa Fluors, donkey anti-chicken 596 and horse anti-mouse 488) for 1 h at RT. We then rinsed slides for 60 min (3×20 min), mounted with Vectashield Mounting Medium H1000 (Vector Laboratories), and examined them on a fluorescence microscope (Leica).

### BrdU incorporation assay

We purchased 5′-Bromo-2′-deoxyuridine (BrdU) from Sigma and dissolved it in double distilled water at 10 mg/ml. Starting at 24 hrs before sacrifice, we injected mice intraperitoneally every 6 hrs with 10 µg BrdU per gram body weight. Following harvest, we embedded tissues in paraffin and labeled them following the BrdU Staining Kit (Zymed Laboratories Inc.) manufacturer's instructions.

### TUNEL assay

We purchased Fluorescein-Frag ELTM DNA Fragmentation Detection Kit (CALBIOCHEM), labeled paraffin-embedded tissue sections following the manufacturer's instructions, and counted the labeled nuclei by using a standard fluorescein filter at 465–495 nm.

### Statistics

We presented the data as the mean ± standard deviation (SD). We made comparisons between groups using a two-sided Student's t test. Differences with P values *P<0.05, **P<0.01, ***P<0.001 were considered significant.

## Results

### Prostate decreased its luminal epithelial cells and secretion function in mice lacking epithelial AR

**Figure 1:**
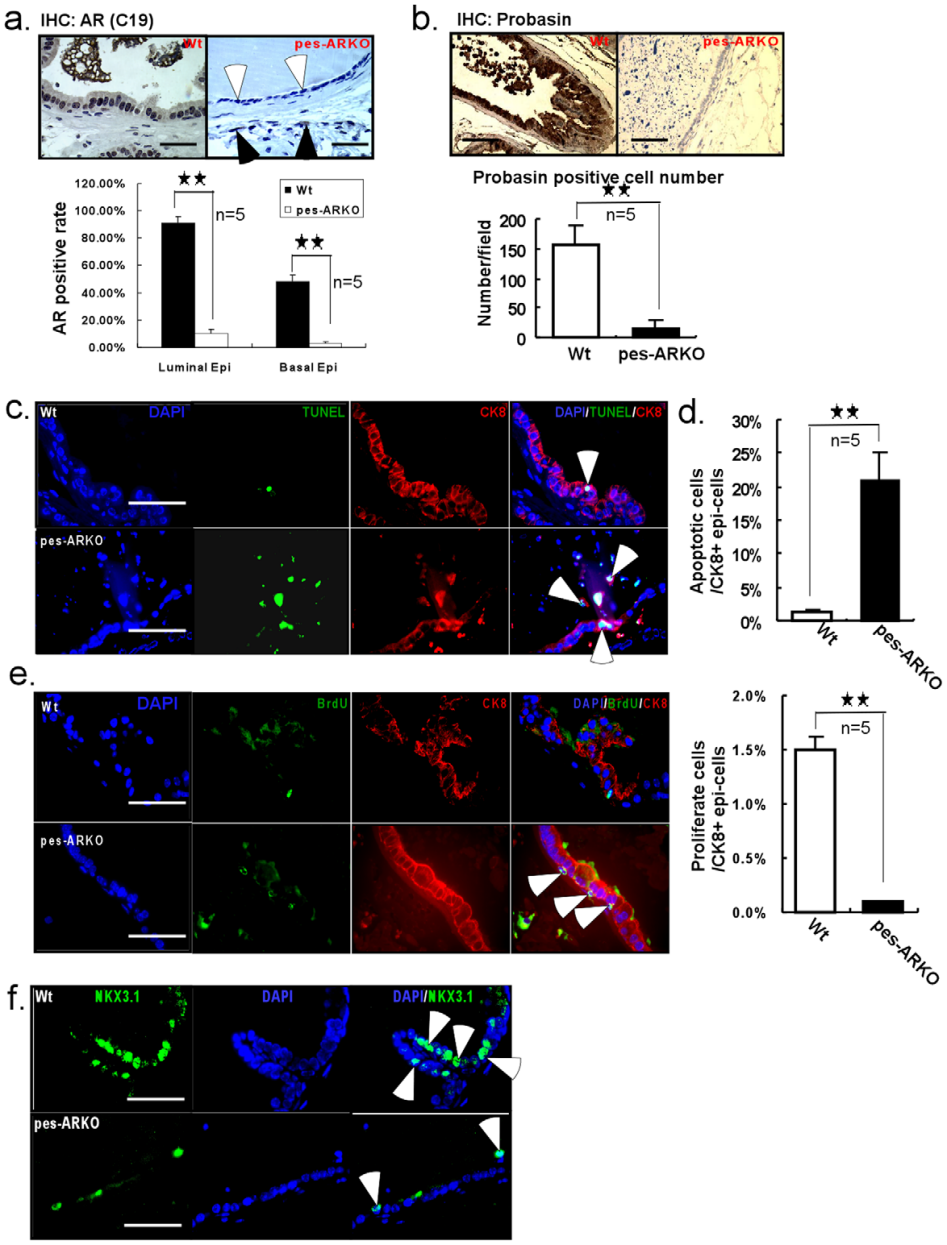
Knockout of AR in the epithelium of mouse prostate led to the lost of functional luminal cells. a) IHC staining showed that the expression of AR was knocked out in the epithelium of the pes-ARKO prostate of 24 weeks old mice (upper panel, white arrows), without affecting the AR expression in the stroma (upper panel, black arrows). AR positive rate in the luminal and basal cells was counted (lower panel). Scale bars, 50 µm. b) The expression of probasin, whose secretion from the murine luminal epithelial cells is dependent on the androgen action, was detected by IHC staining. The decrease of probasin expression (upper panel) and less positive expression (lower panel) indicated the loss of functional epithelium in the pes-ARKO epithelium. Scale bars, 50 µm. c) The double staining of TUNEL and CK8 demonstrated more apoptosis signals from the CK8+ luminal epithelial cells of the pes-ARKO than that of the Wt, apoptotic cell counts were shown in d). Scale bars, 40 µm. e) The double staining of BrdU and CK8. The BrdU positive signal in pes-ARKO epithelium is from CK8 negative cells, but not from CK8 positive cells (as shown by white arrows). There were fewer proliferation cells from the CK8+ luminal epithelial cells of the pes-ARKO than that of the Wt. Scale bars, 40 µm. f) The loss of luminal epithelial cells of pes-ARKO mice was further confirmed by the NKX3.1 staining, which is expressed only in luminal epithelial cells. Scale bars, 40 µm. Data was analyzed with ANOVA and significant P values are indicated by presence of asterisks: ★ (P<0.05), ★★ (P<0.005).

Using flox-cre strategy, we were able to generate the epithelial AR knockout mouse, known as pes-ARKO [17], which had high efficiency of knockout AR (Fig. 1a). AR is gradually deleted in the prostatic epithelium of the ventral, dorsal-lateral lobes, but not significantly in the anterial lobes, which is in agreement with early report [17]. We found pes-ARKO mouse lost its normal function of expressing and secreting probasin (Fig. 1b), an androgen-regulated protein specifically expressed in the differentiated prostate epithelial cells. We also noticed that knockout of epithelial AR led to increased apoptosis in the CK8+ luminal cells (Fig. 1c, 1d), as well as decreased proliferation in the CK8+ luminal cells (Fig. 1e). The expression of homeobox protein NKX3.1, that plays important roles in the maintenance of normal morphogenesis of prostate, was also found to be decreased in the epithelium of pes-ARKO prostate (Fig. 1f).

### Increased proliferation in CK5+ basal epithelial cells in mice lacking epithelial AR

**Figure 2:**
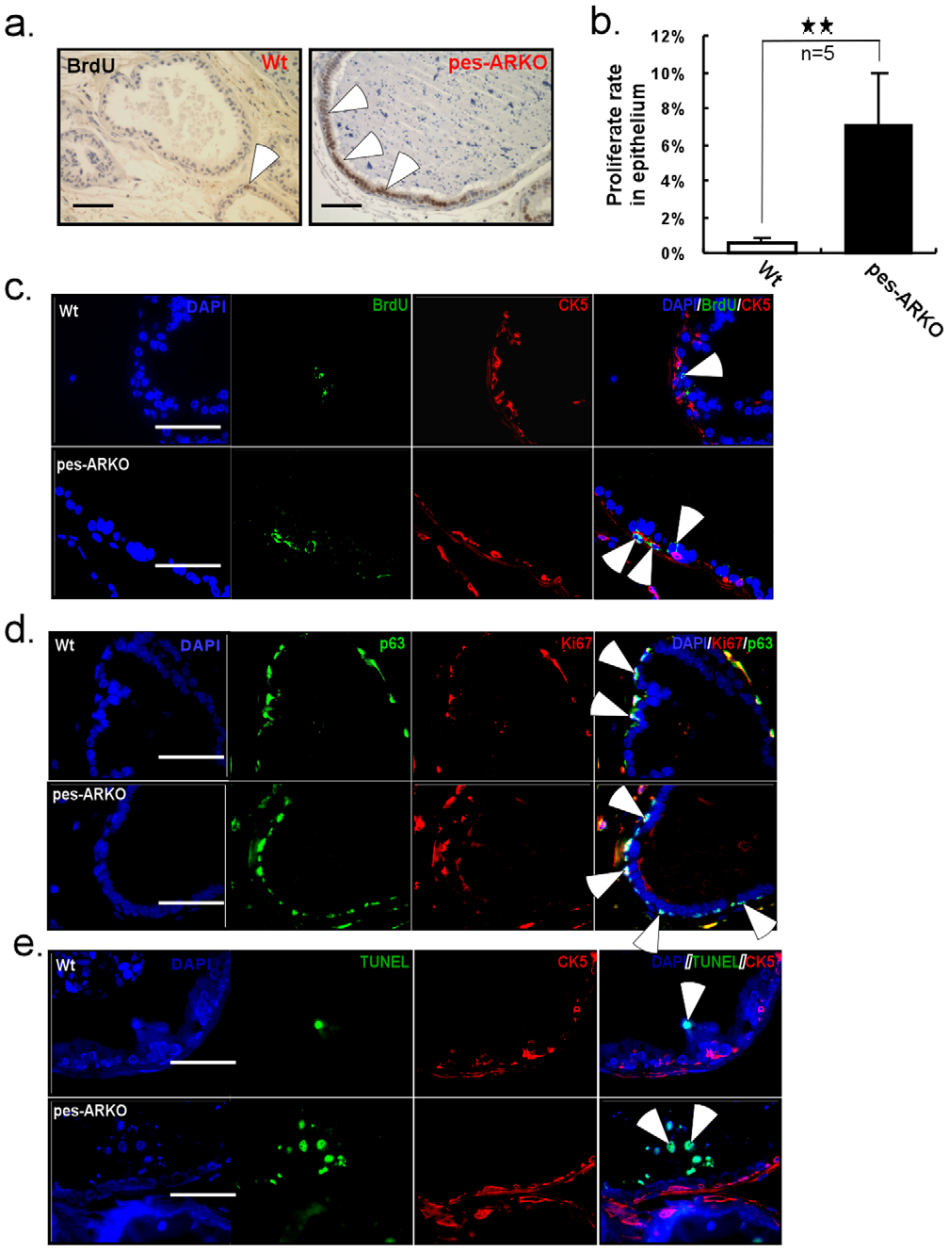
Knockout of AR in the epithelium of mouse prostate induced the proliferation of basal-progenitor cells. a) BrdU IHC staining showed the increased proliferation in AR knockout epithelium of pes-ARKO comparing with that in Wt. Scale bars, 50 µm. b) The difference of the proliferation rate between Wt and pes-ARKO mice was demonstrated. We counted the BrdU positive cell number per 100 epithelial cells. c) Further study by double staining BrdU with basal marker CK5 indicated that the increased proliferation of pes-ARKO epithelium was attributed to basal epithelial cells. d) The proliferation, which was demonstrated by Ki67 expression, in the panel p63+ epithelial cells was also increased in pes-ARKO mice. Scale bars, 40 µm. e) The apoptosis (shown by TUNEL) did not involve CK5+ basal cells in either Wt or pes-ARKO mice. Scale bars, 40 µm.

As shown in Fig. 1a, AR was also knocked out in the CK5+ basal epithelial cells which include stem cells, progenitor cells and intermediate cells [18], even though only half of these basal cells in the wild type (Wt) prostate were AR+ stained. Interestingly, knockout of AR from these parts of the basal cells was sufficient to promote the proliferation of these cells (Fig. 2a, 2b, 2c, 2d). We also found the higher proliferation signals was coincident with the higher expression of progenitor markers, p63 (Fig. 2d) and CD44 (data not shown), suggesting these increased proliferating cells may come from CK5+ basal cells (Fig. 2c). Meanwhile, little apoptotic signal was found in the CK5+ basal cells of both Wt and pes-ARKO prostate (Fig. 2e).

### Epithelial cell population changes in mice lacking epithelial AR

**Figure 3:**
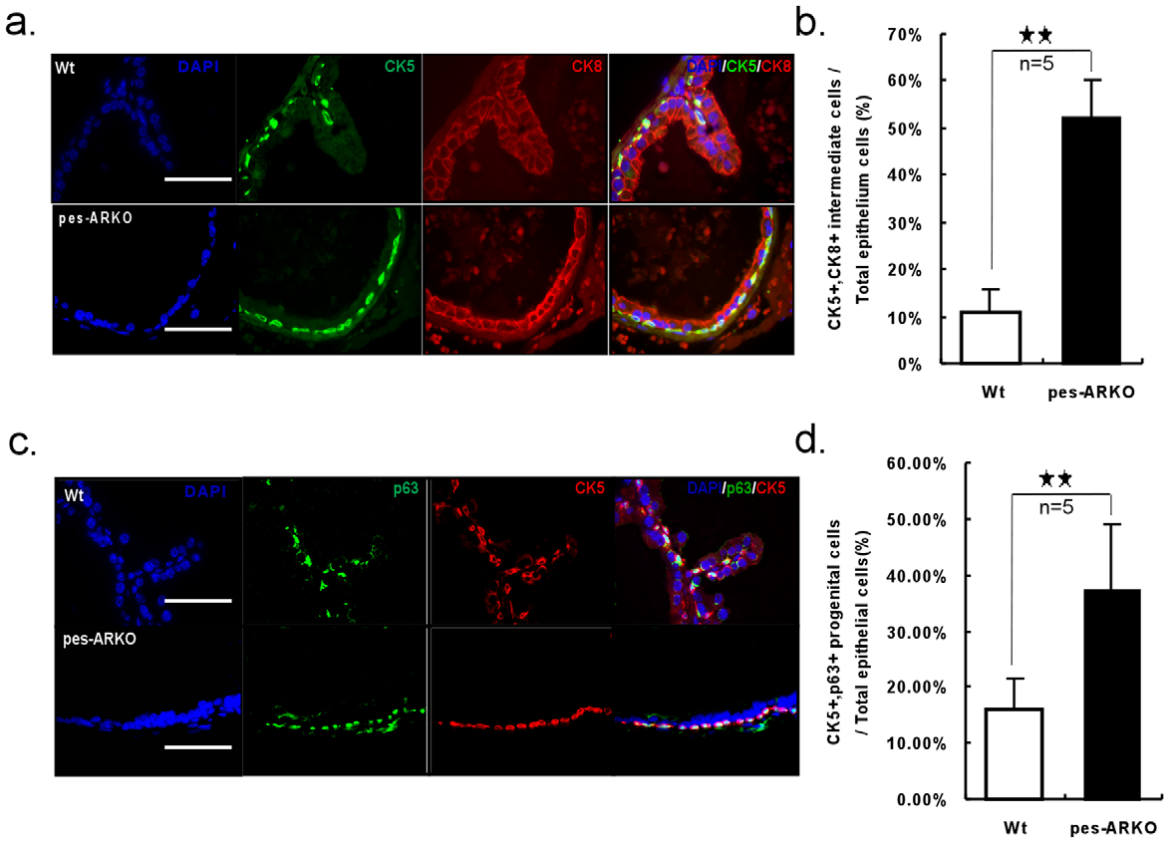
Progenitor cell population expended as the consequence of increased apoptotic CK8+ cells and increased proliferative CK5+ cells in pes-ARKO prostate. a) The CK5 and CK8 double positive intermediate cells were also increased in pes-ARKO mice and were counted in b). Scale bars, 40 µm. c) The CK5 and p63 double positive progenitor cells were increased in pes-ARKO mice and were counted in d). Scale bars, 40 µm.

The increased apoptotic CK8+ luminal cells (Fig. 1) and increased proliferative CK5+ basal cells (Fig. 2) led to the expansion of CK5+/CK8+ intermediate cells (Fig. 3a, 3b) in the epithelium. Interestingly, because these expanded cells are also both p63+ and CK5+, they may therefore be also defined as expanded stem/progenitor cells in the epithelium (Fig. 3c, 3d).

### Altered ductal morphogenesis with decreased ductal branches in mice lacking epithelial AR

**Figure 4:**
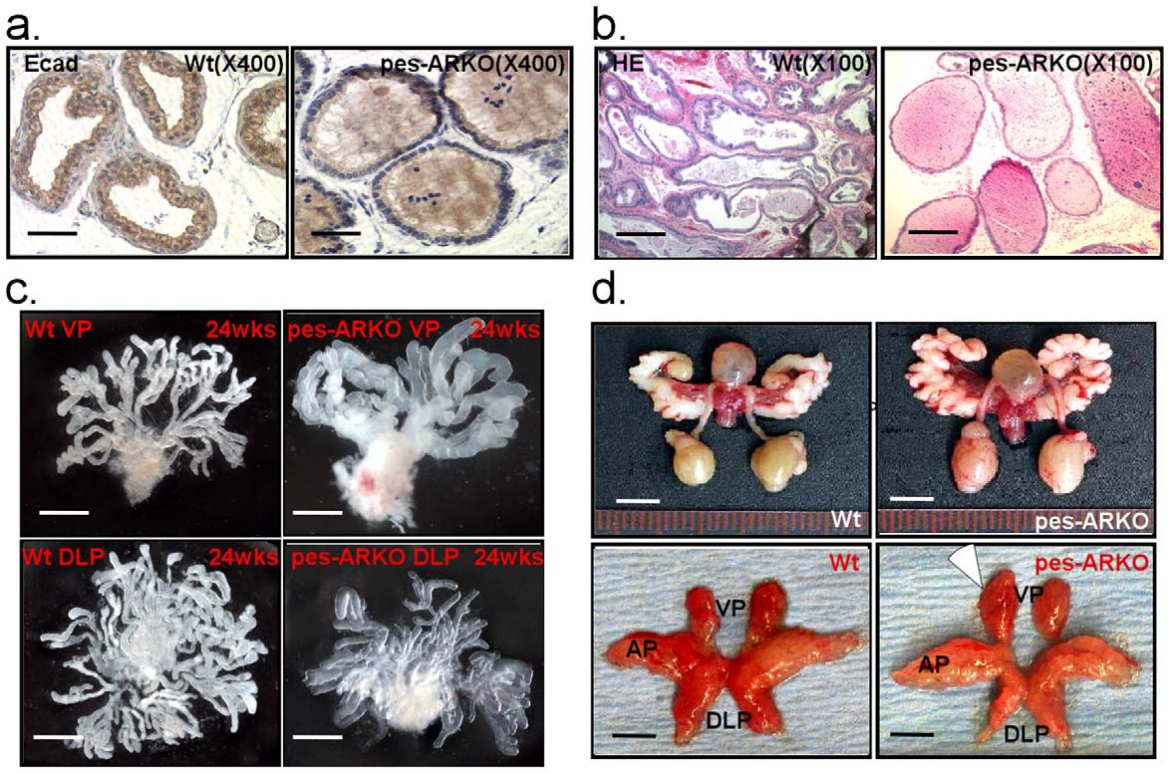
The immature epithelial cells of the pes-ARKO prostate are unable to form normal ductal system. a) The decreased expression of E-cadherin, which is the key molecule of cell-cell tight junction (brown color), in pes-ARKO mice indicated that the loss of luminal epithelium (demonstrated in Fig. 1) in pes-ARKO mice made the epithelium loose. Scale bars, 50 µm. b) H&E staining showed that the lumen of the pes-ARKO mice became round and dilated. Scale bars, 200 µm. c) Using the branching micro-dissection technique, we showed that the lumen of both ventral and dorsal prostate were enlarged, but had fewer branching, especially distal branching loss in ventral prostate of the 24 weeks pes-ARKO mice. Scale bars, 500 µm. d) There is a slight enlargement of the ventral prostate in the 24 weeks pes-ARKO mice from the gross out-look. Upper penal scale bars 5 mm, lower penal 2 mm.

In addition to changes of cell population within epithelium, loss of epithelial AR resulted in decreased E-cadherin expression (Fig. 4a), which might lead to the damaged tight junction and barrier in the epithelium surface that leads to loose epithelium [19]. Using H&E staining (Fig. 4b) and prostate micro-dissection for ductal morphogenesis (Fig. 4c), we found that the prostate lumen of the pes-ARKO mice became round and dilated, folding dismissed, and less branch-points. Notably, at 24 weeks, the pes-ARKO ventral prostates (VPs) showed significantly decreased ductal branches and dilated lumen in VPs and dorsal-lateral prostates (DLPs) (Fig. 4c), and the size of VPs becomes larger as compared to those from Wt mice (Fig. 4d).

### Thinner wall of the lumens with impaired stromal smooth muscle differentiation in mice lacking epithelial AR

**Figure 5:**
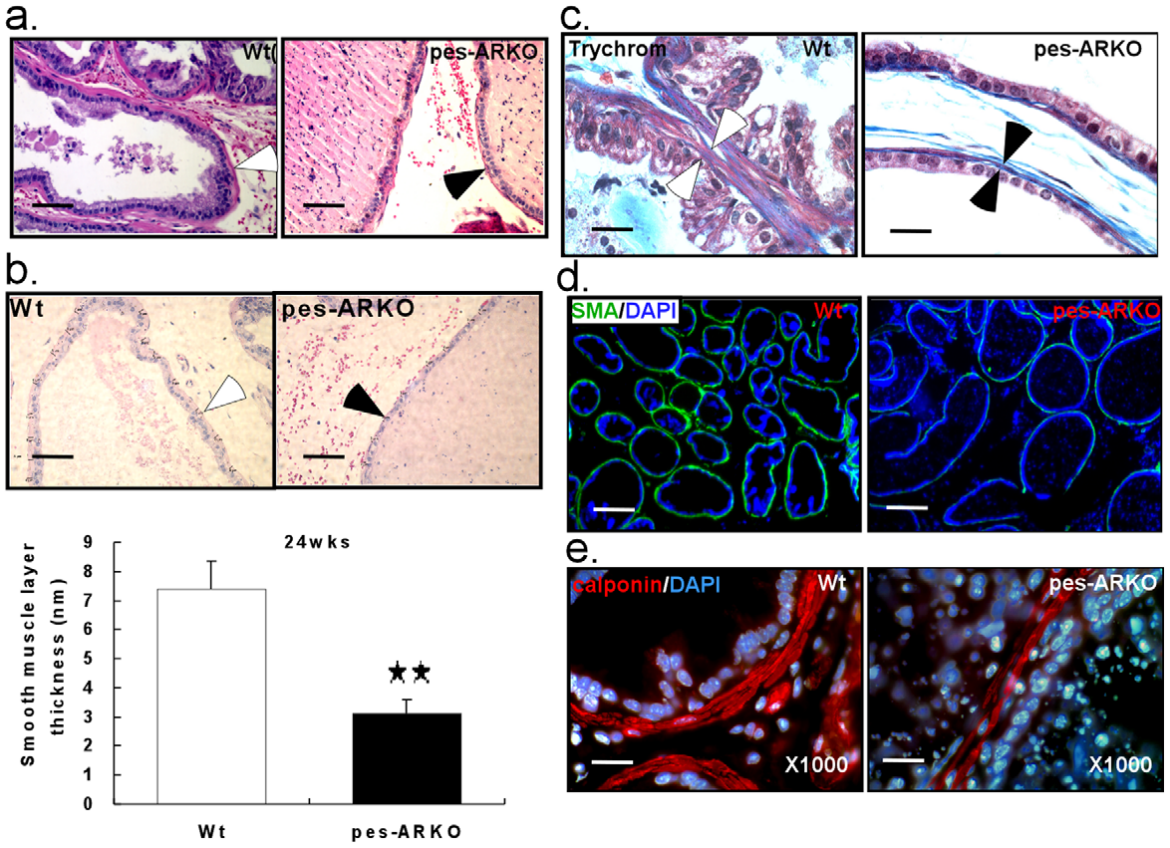
The thickness of the smooth muscle layer, which surrounds the lumen, is significantly thinner in the pes-ARKO prostate than that in the Wt prostate. a) The interesting observation of thinner muscle layer surrounding the lumen of the pes-ARKO mice was indicated in H&E staining, and measured under microscope in b) Scale bars, 50 µm. The average thickness of the smooth muscle layer in pes-ARKO mice was decreased from 7 nm to 3 nm (P<0.01) (lower panel of b). c) Using trichrome staining, the muscle layer (red) of the pes-ARKO mice was thinner than that of the Wt mice. Scale bars, 20 µm. d) Using immunostaining of smooth muscle α-actin (green), we showed that the green circles around the lumen in the Wt samples, were lost or weak in the pes-ARKO samples. Scale bars, 200 µm. e) By staining smooth muscle marker, calponin (red), we also demonstrated the thinner smooth layer around the pes-ARKO epithelium. Scale bars, 20 µm.

In addition to the dilated lumen, we also found thinner stromal smooth muscle layer of VPs in 24 weeks pes-ARKO mice (Fig. 5a and 5b). Using Trichrome staining, we found that the lumen-surrounding stromal smooth muscle (Fig. 5c, between arrows) but not the collagen (blue), was absent in the VPs of pes-ARKO mice. As expected, we found that the mature stromal smooth muscle markers, smooth muscle α-actin (SMA) (Fig. 5d) and calponin (Fig. 5e) were significantly decreased, in the VPs of 24 weeks pes-ARKO mice compared to Wt mice.

### Decreased TGF-β_1_ signals in mice lacking epithelial AR

**Figure 6:**
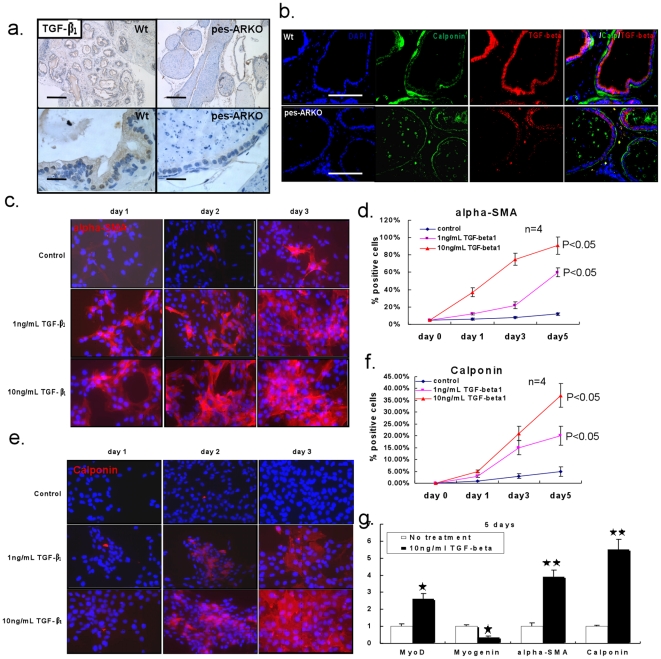
The reduction of the expression of TGF-β_1_ in the pes-ARKO prostate epithelium may cause the thin smooth muscle layer. a) The expression of TGF-β_1_ was investigated by IHC staining (brown color). The decreased expression of TGF-β_1_ was found in pes-ARKO epithelium. Upper penal scale bars 200 µm, lower penal 20 µm. b) By double staining TGF-β_1_ and calponin, we demonstrated that the loss of TGF-β_1_ expression in the epithelium of pes-ARKO mice was coincident with the thinner surrounding smooth muscle layer. Scale bars, 100 µm. c) To evaluate the TGF-β_1_ effect on the differentiation of smooth muscle cells, the primary cultured human prostate stromal cells were treated with different doses of TGF-β_1_. The expressions of smooth muscle α-actin (c and d) and calponin (e and f) were detected following the treatment of 1 to 5 days. g) The genes of MyoD, Myogenin, α-SMA and Calponin, which are involved in the maturation of smooth muscle cells, were investigated after 10 ng/ml TGF-β_1_ treatment.

Since previous data [7], [8] showed that TGF-β_1_ signals may induce the differentiation of prostate stroma that leads to stimulation of the mature of smooth muscle cells, we detected the expression of TGF-β_1_ (Fig. 6a) and TGF-β_1_ downstream signals () by immunohistochemistry (IHC) staining in 24 weeks Wt and pes-ARKO prostate epithelium. The TGF-β_1_ expression and its downstream signals were lower in the epithelium of pes-ARKO mice than those from Wt mice. By double staining TGF-β_1_ and calponin, we demonstrated that the loss of TGF-β_1_ expression (Fig. 6b red) in the epithelium of pes-ARKO mice could be coincident with the thinner layer of the surrounding stromal smooth muscle (Fig. 6b green). Treating the primary cultured human stromal cells (ps-1) with different concentration of TGF-β_1_ for 1 to 5 days, both α-SMA and calponin staining showed that the number of mature stromal smooth muscle cells was significantly dependent on the higher concentration and longer duration of TGF-β_1_ treatment (Fig. 6c, 6d, 6e, and 6f). MyoD and Myogenin expression, which are deeply involved in the stromal smooth muscle maturation and differentiation, was also modulated by TGF-β_1_ stimulation (Fig. 6g).

Altogether, impaired epithelial differentiation by knockout of epithelial AR could lead to poor stromal differentiation that may involve the modulation of TGF-β_1_ signals.

## Discussion

Prostate epithelial growth has been suggested to play important roles for prostate and BPH development. For example, McNeal has proposed an embryonic reawakening hypothesis as a cause for BPH by which the earliest lesion of BPH (that is abundant with stromal smooth muscle cells) could be due to the proliferation of epithelial glandular cells in the transition zone [20]. Our data demonstrated that loss of epithelial AR led to altered epithelial cell proliferation, which in turn could also result in the poor differentiation of stromal smooth muscle cells. It may become an interesting question to ask whether the etiology of BPH could be due to altered epithelial AR signals that lead to altered cell proliferation in epithelium, which then influence surrounding stromal cell differentiation and proliferation. Furthermore, our data showed that the loss of TGF-β_1_ expression (after knockout of epithelial AR) was coincident with the thinner layer of the surrounding smooth muscle, and the number of mature smooth muscle cells was dependent on the higher concentration and longer duration of TGF-β_1_ treatment. Early studies suggested that the major source of TGF-β1 in the normal prostate is from stroma [21], [22]. However, other studies also reported that TGF-β_1_ expression is stained in both normal epithelial and stromal cells [23], [24], and demonstrated that TGF-β_1_ was expressed by primary cultured normal human prostate epithelial cells [25], [26], BPH-1 and NRP-152 epithelial cell lines [27]. The increased epithelial expression of TGF-β_1_ was observed coincidentally with androgen peak during prostate development [28]. Another report also found that TGF-β_1_ mainly localized in the epithelium of dorsal and ventral lobes of prostate [29]. Recently, one significant paper indicated that overexpression of TGF-β_1_ in the epithelium of prostate by transgenic mouse technique may induce fibroplasia and collagenous micronodules in stroma [30]. Here we demonstrated, by our unique model, that lost AR in mouse epithelium gradually resulted in immature development of prostate epithelium and ductal morphogenesis, which decrease generating TGF-β_1_ in the epithelium. Whether this may help us to develop a potential therapeutic approach by either targeting epithelial AR or its downstream target-TGF-β_1_ for treatment of BPH, may become another interesting question to ask in the future.

The increased apoptosis in epithelial CK8+ luminal cells and increased proliferation in epithelial CK5+ basal cells leads to the expansion of p63+/CK5+ progenitor populations and CK5+/CK8+ intermediate cells. These cell population changes confirm the existence of intermediate cells that are phenotypically intermediate between basal and luminal cells, and more importantly, as these intermediate cells are also p63+ (a progenitor cell marker), it may also indicate that basal cells and luminal cells are hierarchically related, basal cells may represent the progenitors of luminal cells [31]–[33]. Since loss of epithelial AR resulted in impaired ductal morphogenesis and enlargement of the VP gland, this may also suggest that progenitor cells (and their original stem cells) may be responsible for tissue homeostasis of epithelial tissues with diverse architectural design and physiology [34]. This further confirms that epithelial AR may not only be required for epithelial cell differentiation, but also function as a proliferation suppressor for epithelial CK5+/CK8+ intermediate cells and a survival factor for epithelial CK8+ luminal cells. These two opposite roles of the AR in different epithelial cells appear to contribute significantly to cellular homeostasis in the prostate, although the underlying mechanisms remain to be elucidated.

It has been proposed that cancer may come from neoplastic transformation of stem cells [35], which could then generate progenitor cells, and progress sequentially into CK5-positive-basal cells, CK5/CK8-positive-basal intermediate cells, and then CK8-positive luminal cells [36]. We believe this course of prostate cancer cell differentiation is also controlled under androgen/AR regulation, which just mimics the normal epithelial cell hierarchical lineage.

Similar to previously reported [37], [38], we also confirmed the AR regulation roles in the differentiation of tumor cells by specifically knocking out epithelial AR from the transgenic adenocarcinoma of the mouse prostate (TRAMP) that could spontaneously developed prostate cancer. We observed that increased proliferating cancer stem/progenitor cells (Fig. S2a), and expanded cancer stem/progenitor populations, defined as p63+/CK5+ double positive (Fig. S2b), Sca-1+ (Fig. S2c), or CD133+ (Fig. S2d), were significantly different in pes-ARKO TRAMP comparing with the Wt TRAMP. Our studies also support the existence of the stem/progenitor cell populations in the prostate cancer, and we believe that the differentiation of the cancer stem/progenitor cells could be also modulated by androgen/AR signals so that AR could function differently in cancer stem/progenitor cells and differentiated luminal-like cancer cells [38]. The opposite functions of the epithelial AR in different epithelial cells could then affect prostate cancer progression in TRAMP mice by favoring survival of differentiated tumor epithelium while suppressing proliferation of epithelial-basal intermediate cells.

### Conclusion

Taken together, using this pes-ARKO mouse model, we conclude that epithelial AR plays essential yet diverse roles in development and adult homeostasis of the prostate gland, and epithelial AR plays important roles in the prostate stromal development via the regulation of TGF-β_1_ signal.

## Supporting Information

Figure S1 **The TGF-β_1_ signaling was decreased in the epithelium of pes-ARKO prostate.** Using IHC staining, we detected the difference of TGF-β_1_, TGF-β RII, Smad2/3 and Smad4 protein levels between Wt and pes-ARKO ventral prostate (upper). Then, we separated the ventral epithelium by laser capture microdissection, and checked the relative mRNA levels of TGF-β_1_ and TGF-β RII in Wt and pes-ARKO samples (lower). The results from both assay indicated that TGF-β_1_ signaling in AR knockout prostate epithelium is impaired. Scale bars, 50 µm.
(TIF)

Figure S2 **The increased proliferating cancer stem/progenitor cells, and expanded cancer stem/progenitor populations in pes-ARKO TRAMP.** a) The proliferation of progenitor cells in the pes-ARKO-TRAMP mice was increased. The double staining of the progenitor marker p63 with the proliferation marker Ki67 indicated that the proliferation of progenitor cells in the pes-ARKO-TRAMP mice was increased compared to Wt-TRAMP mice and castrated TRAMP mice. b) CK5 (red) and p63 (green) double staining. The CK5+/p63+ progenitor population was increased in 12 weeks old pes-ARKO TRAMP prostate compared to same aged Wt TRAMP mice. Scale bars, 40 µm. c) and d) Increased progenitor/stem cell population in pes-ARKO TRAMP mice as indicated with Sca-1 and CD133 expression compared to Wt-TRAMP mice and castrated TRAMP mice using flow cytometry method.
(TIF)
