# Supplementary figures and images for: Increased CK5/CK8-Positive Intermediate Cells with Stromal Smooth Muscle Cell Atrophy in the Mice Lacking Prostate Epithelial Androgen Receptor
Source: PLoS One. 2011 Jul 6;6(7):e20202. doi: 10.1371/journal.pone.0020202 (PMC3130731; doi:10.1371/journal.pone.0020202)

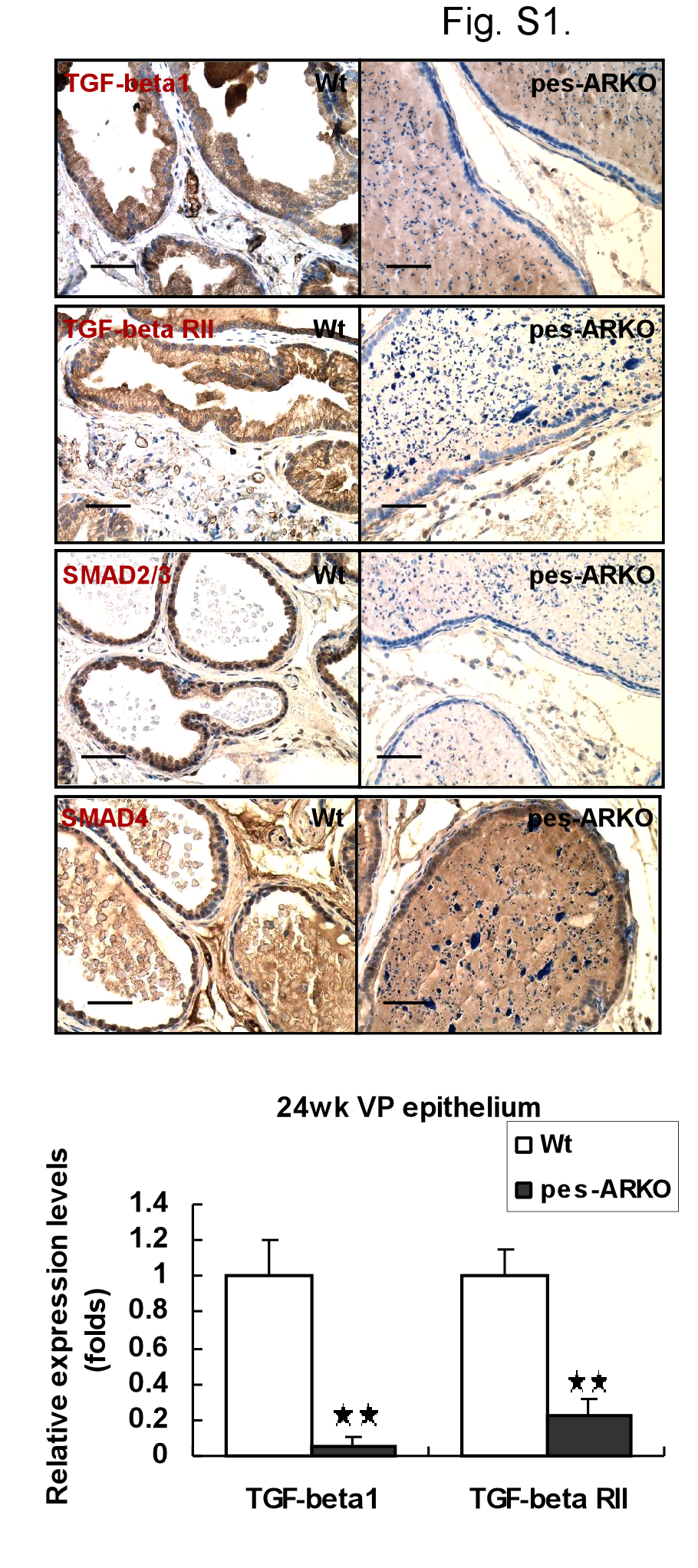

Supplement: Figure S1 — The TGF-β1 signaling was decreased in the epithelium of pes-ARKO prostate. Using IHC staining, we detected the difference of TGF-β1, TGF-β RII, Smad2/3 and Smad4 protein levels between Wt and pes-ARKO ventral prostate (upper). Then, we separated the ventral epithelium by laser capture microdissection, and checked the relative mRNA levels of TGF-β1 and TGF-β RII in Wt and pes-ARKO samples (lower). The results from both assay indicated that TGF-β1 signaling in AR knockout prostate epithelium is impaired. Scale bars, 50 µm. (TIF) [file pone.0020202.s001.tif]

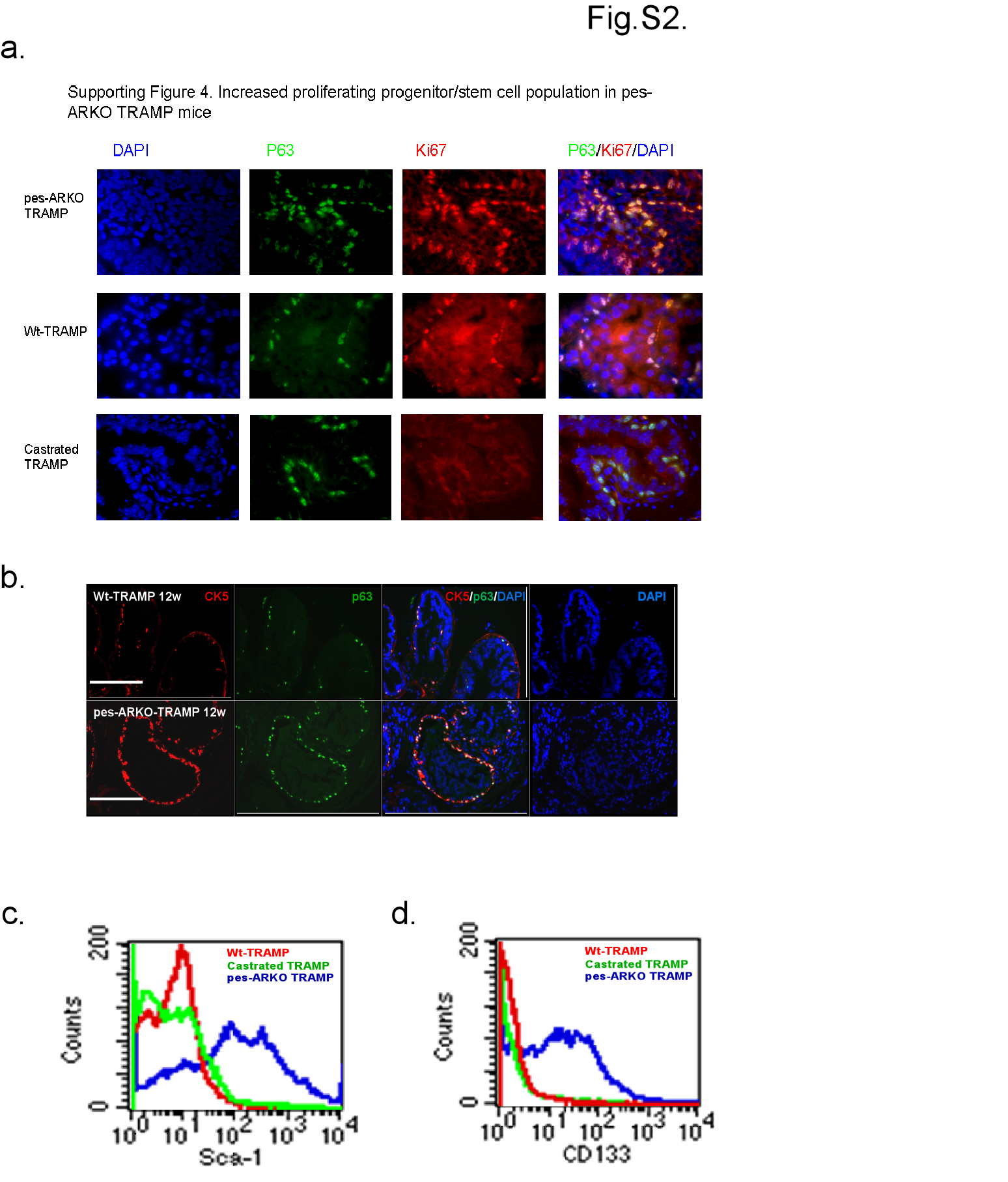

Supplement: Figure S2 — The increased proliferating cancer stem/progenitor cells, and expanded cancer stem/progenitor populations in pes-ARKO TRAMP. a) The proliferation of progenitor cells in the pes-ARKO-TRAMP mice was increased. The double staining of the progenitor marker p63 with the proliferation marker Ki67 indicated that the proliferation of progenitor cells in the pes-ARKO-TRAMP mice was increased compared to Wt-TRAMP mice and castrated TRAMP mice. b) CK5 (red) and p63 (green) double staining. The CK5+/p63+ progenitor population was increased in 12 weeks old pes-ARKO TRAMP prostate compared to same aged Wt TRAMP mice. Scale bars, 40 µm. c) and d) Increased progenitor/stem cell population in pes-ARKO TRAMP mice as indicated with Sca-1 and CD133 expression compared to Wt-TRAMP mice and castrated TRAMP mice using flow cytometry method. (TIF) [file pone.0020202.s002.tif]
